# Mini-Thoracotomy Versus Subxiphoid Pericardial Window: Two Cases of Hemodynamically Significant Pericardial Effusions

**DOI:** 10.7759/cureus.110065

**Published:** 2026-06-01

**Authors:** Daniela H Rodriguez Manrique, Efren Buitrago

**Affiliations:** 1 Surgery, Ross University School of Medicine, Miami, USA; 2 Cardiovascular and Thoracic Surgery, Jackson Memorial Hospital, Miami, USA

**Keywords:** cardiac tamponade, pericardial effusion, pericardial window, surgical approach, surgical management

## Abstract

Pericardial effusions can progress to cardiac tamponade, a life-threatening condition that requires immediate management. Although the first-line treatment for cardiac tamponade is pericardiocentesis, a surgical approach becomes necessary to create a pericardial window in cases of failure to drain, recurrence, or loculated effusions or in unsafe percutaneous procedures. We present two cases of hemodynamically significant pericardial effusions treated with different techniques to emphasize the determinants in choosing the most appropriate surgical approach. The first case is a 46-year-old male patient with pneumonia and a progressively increasing pericardial effusion with early signs of tamponade who underwent a left anterior mini-thoracotomy for the creation of a pericardial window. The second case is a 50-year-old male patient with a past medical history of bilateral lung transplantation who developed a loculated pericardial effusion and signs of tamponade. Because of previous thoracic surgery and an unsafe percutaneous access, a subxiphoid window with xiphoidectomy and a small wedge sternotomy was performed. The patients in both cases showed resolution of tamponade physiology and symptomatic relief after surgical drainage. Pathologic evaluation in both patients revealed fibrous pericarditis, with no evidence of malignancy or infection. These two cases showcase that the choice of surgical method for pericardial window depends on patient-specific factors, which include previous thoracic surgery, percutaneous accessibility, and the nature of pericardial effusion. Thus, careful operative planning is key to optimize outcomes and minimize procedural risks.

## Introduction

A pericardial effusion is the accumulation of fluid within the pericardial sac surrounding the heart [1]. Its diverse causes include pericarditis, malignancy, renal failure, infection, trauma, and postoperative states. Patients can present with small to moderate or moderate to large effusions, which can alter the course of management. In a case with small to moderate pericardial effusion, the patient can remain asymptomatic, and procedural management may not be needed. However, in a large or rapid accumulation of fluid, symptoms can develop along with complications such as cardiac tamponade, which warrant an intervention [1,2].

Cardiac tamponade is a life-threatening condition which encompasses impaired cardiac filling and reduced cardiac output [1,2]. Cardiac tamponade occurs when the pressures in the pericardial sac are greater than the pressures in the heart chambers. This difference in pressures affects diastolic filling and consequently leads to chamber collapse along with reduced cardiac output. The diagnostic imaging of choice for cardiac tamponade is a transthoracic echocardiogram (TTE). An early physiological sign of tamponading is right atrial collapse, which can be visible in an echo. As the tamponade progresses, right ventricular diastolic collapse becomes apparent in the same imaging tool [2,3]. Clinical findings may be present which could corroborate the diagnosis of cardiac tamponade. The classic clinical findings include hypotension, jugular venous distention, and muffled heart sounds. But the presence of these can vary depending on the rate of fluid accumulation [1].

Pericardiocentesis is the intervention of choice for most patients with moderate to severe pericardial effusions. This approach in management is frequently effective to relieve symptoms and to restore hemodynamic stability [1,3]. Yet, surgical treatment with the creation of a pericardial window may be necessary in certain cases of failed pericardiocentesis and recurrent or loculated effusions and in patients with unsafe percutaneous access due to prior thoracic procedures [4].

There are currently different surgical approaches that can be employed to create a pericardial window. The most common approaches are anterior mini-thoracotomy and subxiphoid access [4,5]. Other approaches include anterior parasternal incision and video-assisted thoracoscopic surgery (VATS) [6,7]. The surgical approach should be selected depending on the patient's anatomy, effusion pathophysiology, the patient's comorbidities, and the surgeon's expertise.

We present two cases of hemodynamically significant pericardial effusions that serve as an educational comparison that highlights patient-specific determinants in the operative planning process for the creation of a pericardial window. 

## Case presentation

Case 1

The first patient is a man in his 40s with a past medical history of type 2 diabetes mellitus and heavy tobacco use who presented to another institution for pressure-like chest pain. An ST-segment elevation myocardial infarction (STEMI) alert was activated, and the patient was transferred to our institution where cardiology evaluated him and canceled the alert after reviewing the test results. The patient had negative cardiac biomarkers, a normal brain natriuretic peptide (BNP) level, and no signs of ST elevation in his electrocardiography (ECG), which did not correlate with a STEMI diagnosis. He was admitted to the hospital for leukocytosis and pneumonia. A TTE was done in the hospital, revealing a small- to moderate-sized pericardial effusion without evidence of tamponade.

**Figure 1:**
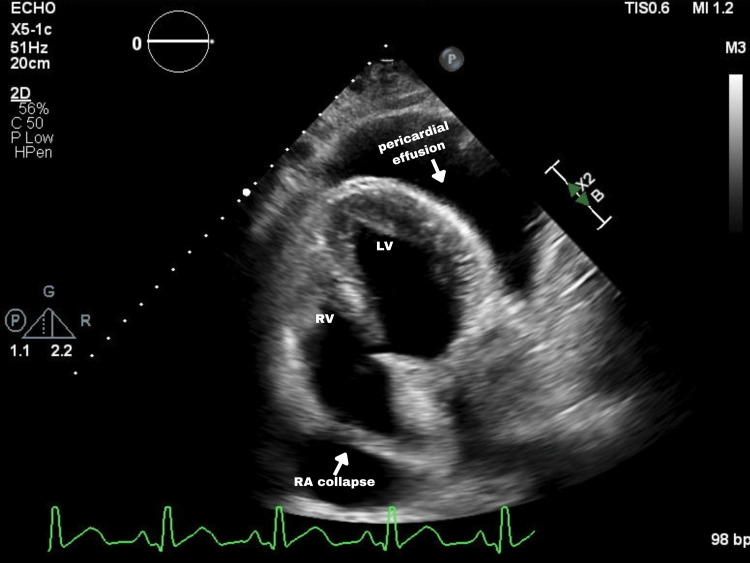
Transthoracic echocardiogram demonstrating pericardial effusion with right atrial collapse consistent with early tamponade physiology in Case 1. RA: right atrium; RV: right ventricle; LV: left ventricle

A TTE was repeated on day 6 from hospital admission, showing the progression of the size of the pericardial effusion. Two days later, another TTE was performed, showing the further progression of the pericardial effusion and evidence of early signs of tamponade physiology (see Figure 1). There were evident right atrial collapse and increased respiratory variation in mitral inflow velocities, as reported by the radiologist. ECG revealed diffuse ST elevations, which were interpreted as possible pericarditis or early repolarization.

The considered diagnoses were STEMI versus pericarditis versus pericardial effusion progression to cardiac tamponade. Due to non-elevated cardiac biomarkers, a STEMI was ruled out. Considering the diffuse ST elevations on ECG, pericarditis or early repolarization was considered. However, the TTE showing a pericardial effusion with right atrial collapse supported the diagnosis of cardiac tamponade.

The patient was recommended to undergo pericardiocentesis by interventional cardiology. Pericardiocentesis was attempted on day 7, but it was unsuccessful, with no fluid aspirated following percutaneous access of the pericardium using imaging guidance. Even though the effusion was neither loculated nor posterior, drainage could not be achieved. Cardiothoracic surgery was then consulted to manage the patient.

On day 9, the patient underwent a left anterior mini-thoracotomy with the creation of a window into the pericardial space under general anesthesia with intermittent lung ventilation holds. A large volume of hemorrhagic fluid was drained. Pericardial fluid and tissue were sent for microbiologic and pathologic examination.

Postoperatively, the patient showed signs of improvement with relieved chest pain and shortness of breath. He underwent a follow-up TTE on postoperative day 2, which showed trace effusion without evidence of tamponade physiology. The cytology examination did not show malignancy in the pericardial effusion, and the cultures were negative. The pathology examination on the pericardial tissue showed fibrous pericarditis.

Case 2

The second patient is a man in his 50s with a history of recurrent pleural effusions status post bilateral lung transplantation. He presented to our institution with increasing shortness of breath, generalized edema, and anasarca.

**Figure 2:**
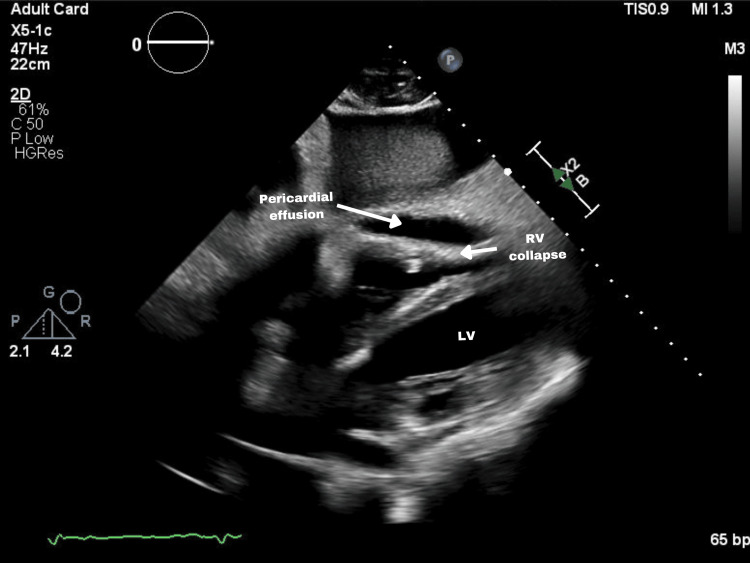
Transthoracic echocardiogram demonstrating pericardial effusion with right ventricular free wall collapse indicating tamponade physiology in Case 2. RV: right ventricle; LV: left ventricle

On day 3 from hospital admission, a TTE revealed an increase in the size of a previously documented loculated pericardial effusion along with a new presentation of partial compression of the right ventricle. Two days later, another TTE was done demonstrating the progression of the pericardial effusion and right ventricular diastolic collapse, consistent with established cardiac tamponade physiology (see Figure 2).

The interventional cardiology service was consulted for possible percutaneous pericardiocentesis. Pericardiocentesis was deferred due to unsafe percutaneous access. The patient was then referred to cardiothoracic surgery for surgical intervention.

Given the history of a bilateral lung transplant, a standard anterior thoracotomy was considered high risk due to expected dense adhesions in the mediastinal region. On day 6, a pericardial window through a subxiphoid approach was performed. There was extensive fibrosis in the subxiphoid and retrosternal areas. A limited, wedge-shaped distal sternectomy was necessary, as well as a xiphoidectomy, for adequate exposure of the pericardial space.

During the operation, a serosanguineous pericardial effusion was drained. There was also an observable thickened and inflamed pericardium with pale fibrotic epicardium, consistent with chronic inflammation. An intraoperative TEE confirmed complete drainage of the pericardial effusion with improved right atrial and right ventricular filling.

The patient showed clinical improvement postoperatively with decreased shortness of breath. A TTE performed on postoperative day 1 revealed trace amounts of pericardial effusion with preserved systolic function. Microbiologic cultures were negative for growth. Cytologic examination of the pericardial fluid for malignancy was negative. Histopathologic examination of the pericardial tissue revealed fibrous pericarditis. The resected xiphoid and sternal tissue did not show pathologic changes.

**Figure 3:**
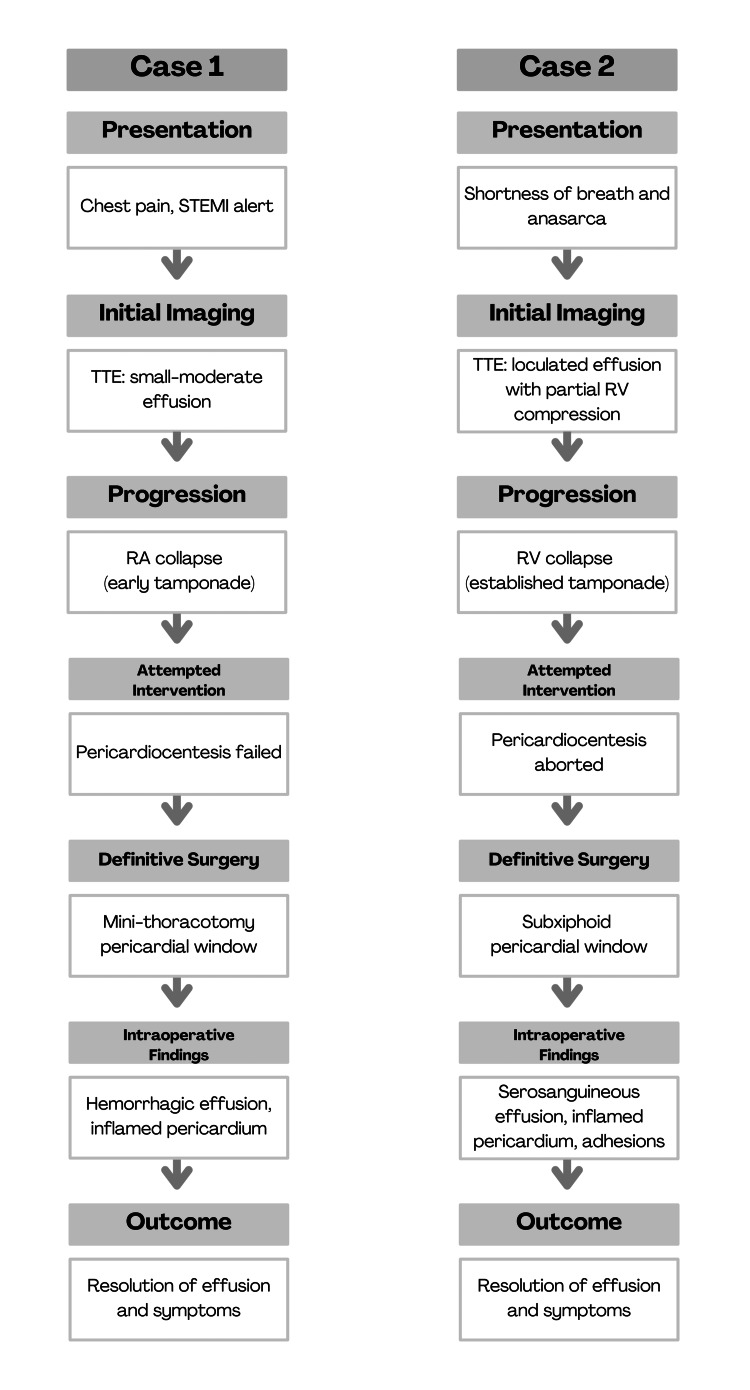
Comparative timeline of patient determinants of surgical approach planning for the creation of a pericardial window. STEMI: ST-segment elevation myocardial infarction; TTE: transthoracic echocardiogram; RA: right atrium; RV: right ventricle

To facilitate the comparison of these cases, Figure 3 and Table 1 summarize the operative decision-making process and the key surgical findings. 

**Table 1:** Comparative clinical and operative characteristics of the two patients undergoing the creation of pericardial windows. RA: right atrium; RV: right ventricle

Variable	Case 1	Case 2
Effusion	Hemorrhagic	Serosanguineous
Tamponade severity	Early	Established
Echo finding	RA collapse	RV collapse
Percutaneous access	Failed drainage	Unsafe access
Surgical approach	Left anterior mini-thoracotomy pericardial window	Subxiphoid pericardial window with xiphoidectomy and distal wedge sternectomy
Prior thoracic surgery	No	Bilateral lung transplant
Intraoperative complexity	Standard access	Dense adhesions
Pathology	Fibrous pericarditis	Fibrous pericarditis

## Discussion

Pericardial effusions can progress to cardiac tamponade when an increase in intrapericardial pressure hinders cardiac filling and reduces cardiac output. The hemodynamic compromise depends on the volume of fluid, the rate of accumulation, and the pericardial compliance. Echocardiograms are essential to the diagnosis of pericardial effusion and its progression to cardiac tamponade. Right atrial collapse represents an early physiological sign, which is often followed by right ventricular diastolic collapse as the tamponade progresses [2,3]. These imaging findings along with the clinical features of cardiac tamponade, such as hypotension, jugular venous distention, and muffled heart sounds, guide the urgency of intervention.

Pericardiocentesis remains the first-line treatment for most patients with significant pericardial effusion [1,3]. However, surgical drainage via pericardial window is indicated in cases of failed pericardiocentesis and recurrent or loculated effusions and in patients with prior thoracic surgery where percutaneous access may be limited or unsafe [4]. In patients with cardiac tamponade requiring surgical intervention, the perioperative process becomes essential to patient outcome. The induction of general anesthesia can lead to hemodynamic collapse because of decreased sympathetic tone. Thus, these patients are prepped and draped while they are awake. This is done to reduce the time between the induction of anesthesia and rapid decompression of the pericardial space during surgical drainage, thereby reducing hemodynamic instability during the procedure.

In Case 1, pericardiocentesis was unsuccessful despite a large effusion, prompting surgical intervention. An anterior mini-thoracotomy provided excellent exposure, allowing the effective drainage of a hemorrhagic effusion. This approach is often preferred when a larger pericardial window is required, as it enables the direct visualization and complete evacuation of non-loculated effusions [4,5].

In contrast, Case 2 highlights the challenges associated with prior thoracic surgery. The patient's history of bilateral lung transplantation resulted in significant mediastinal adhesions. This rendered percutaneous access unsafe, increasing the risk of a standard thoracotomy. A subxiphoid approach was therefore selected to avoid re-entry through previous thoracic incisions. Despite this access choice, dense adhesions were encountered in the mediastinum, requiring a wedged resection of the distal sternum to achieve adequate exposure. This case underscores the importance of operative flexibility and the effect of altered anatomy on surgical planning [4-6].

The choice between mini-thoracotomy and subxiphoid approaches depends on multiple factors, including prior surgical history, characteristics of the effusion, and the need for diagnostic sampling, as visualized in Table 2. A mini-thoracotomy provides better visualization of the pericardium and the ability to create a larger pericardial window. Yet, this approach requires assistance from the anesthesia team to hold ventilation at different times throughout the procedure, is more invasive, and has a greater risk of infection due to pleural contamination [8]. The subxiphoid approach is generally less invasive and avoids thoracotomy, representing less risk for the patient. However, exposure can be limited in patients with prior surgery or significant fibrosis in the mediastinum or the thorax [4-6].

**Table 2:** Comparison of surgical approaches used for pericardial window creation.

Surgical approach	Mini-thoracotomy	Subxiphoid
Used in case	Case 1	Case 2
Indication	Failed pericardiocentesis	Prior thoracic surgery and limited percutaneous access
Access	Left anterior thoracotomy (intercostal space)	Subxiphoid incision with xiphoidectomy
Visualization	Excellent exposure of the pericardium	Moderate, may be limited by adhesions and anatomy
Advantages	Larger window, direct visualization, and effective drainage of non-loculated effusions	Less invasive, avoids thoracotomy, useful in re-operative chest or high-risk patients
Limitations	Requires intermittent ventilation holds by the anesthesia team, more invasive	Limited exposure, technically challenging in patients with fibrosis or prior thoracic surgeries
Intraoperative considerations	Straightforward access with no major technical challenges reported	Required extension to limited wedge sternectomy due to dense adhesions and retrosternal fibrosis
Ideal patient profile	Patients without prior thoracic surgery requiring definitive drainage after a failed percutaneous approach	Patients with prior thoracic surgery or altered anatomy where thoracotomy is less favorable

Both patients in this series demonstrated favorable outcomes following surgical drainage, with the resolution of tamponade physiology and no evidence of infection. Pathologic findings of fibrous pericarditis in both cases suggest an underlying chronic inflammatory process.

The hemorrhagic effusion encountered intraoperatively in Case 1 raised concerns for underlying malignancy or inflammatory process. On the other hand, the serosanguineous effusion of Case 2 was consistent with the patient's presentation of volume overload and chronic disease. Although cytology for Case 1 was negative for malignancy, the hemorrhagic nature of the effusion highlights the important role of routine pathological analysis to rule out high-risk causes and guide prognosis. 

This comparison highlights that prior thoracic surgery is a key determinant in choosing a subxiphoid access for a pericardial window. Meanwhile, a failed percutaneous drainage favors a mini-thoracotomy approach.

## Conclusions

These cases demonstrate that surgical management of hemodynamically significant pericardial effusions may be influenced by patient anatomy, prior thoracic intervention, and the feasibility of percutaneous drainage. Both surgical approaches observed in this report, mini-thoracotomy and subxiphoid, can be useful for the creation of pericardial windows when selected appropriately. Thus, planning a surgical approach tailored to each patient's unique factors is essential to optimize the resolution of tamponade physiology. 
